# Combination chemotherapy with sintilimab for treatment of a male patient with primary pulmonary choriocarcinoma: a case report and literature review

**DOI:** 10.3389/fimmu.2025.1523316

**Published:** 2025-01-23

**Authors:** Xiaojing Liang, Guoqing Xu, Jisong Zhang, Li Xu, Liangliang Dong, Xiaoyue Wang, Weidong Han, Enguo Chen

**Affiliations:** ^1^ Department of Respiratory Medicine, Sir Run Run Shaw Hospital, Zhejiang University, School of Medicine, Hangzhou, Zhejiang, China; ^2^ Department of Pulmonary and Critical Care Medicine, National Regional Medical Center, Zhejiang University Sir Run Run Shaw Alaer Hospital, Alaer, Xinjiang, China; ^3^ Department of Medical Oncology, the Cancer Hospital of the University of Chinese Academy of Sciences (Zhejiang Cancer Hospital), Hangzhou, Zhejiang, China

**Keywords:** primary pulmonary choriocarcinoma, chemotherapy, immune checkpoint inhibitors, programmed death-ligand 1, human chorionic gonadotropin

## Abstract

Primary pulmonary choriocarcinoma (PPC) is an extremely rare malignant cancer in men with no clinical guidelines for the treatment. Cytotoxic chemotherapy has a limited effect on the prognosis of PPC. Recently, immune checkpoint inhibitors (ICIs) have shown promising treatment effectiveness. A literature review revealed that a combination of chemotherapy and ICIs could prolong the survival time of PPC patients. Herein, we report a 67-year-old man with a cough, expectoration, and blood in the sputum, presenting with a mass in the lower lobe of the right lung. The hematoxylin and eosin staining and immunohistochemical profile of the primary lesion showed features of choriocarcinoma, accompanied by elevations in serum human chorionic gonadotropin (HCG) levels. Programmed death-ligand 1 (PD-L1) expression was positive (70%) in the 22C3 assay. He received a combination of cisplatin, etoposide, paclitaxel, and sintilimab. Four months after the treatment began, chest computed tomography (CT) showed significant mass reduction. Therefore, ICIs may be a promising therapy option for patients with PPC.

## Introduction

Choriocarcinoma is a malignant trophoblastic tumor that produces human chorionic gonadotropin (HCG) (1). Most cases of choriocarcinoma stem from the chorionic villi of the uterus following a pregnancy. As a non-gestational choriocarcinoma, primary pulmonary choriocarcinoma (PPC) is an extremely rare cancer in men, and we identified only 36 cases with specific information from published English literature to date (**Supplementary Table 1**). Due to its rarity, there is currently no established standard treatment for PPC. Previous case reports confirmed that surgery combined with chemotherapy seems to be the most appropriate treatment (2). The chemotherapy regimens commonly used in PPC treatment were mostly based on female choriocarcinoma or male germ cell tumors, including the PEB regimen, EMA-CO regimen, EMA-EP regimen, and so on (2). Immune checkpoint inhibitors (ICIs) are promising for the treatment of choriocarcinoma since most choriocarcinoma specimens exhibit diffuse and intense PD-L1 immunoreactivity in syncytiotrophoblasts (3). Some male primary choriocarcinomas have been reported to respond to PD-1/PD-L1 blockade therapy (4, 5). Herein, we report a patient who received chemotherapy combined with sintilimab and remains in remission now for 12 months.

## Case report

**Figure 1:**
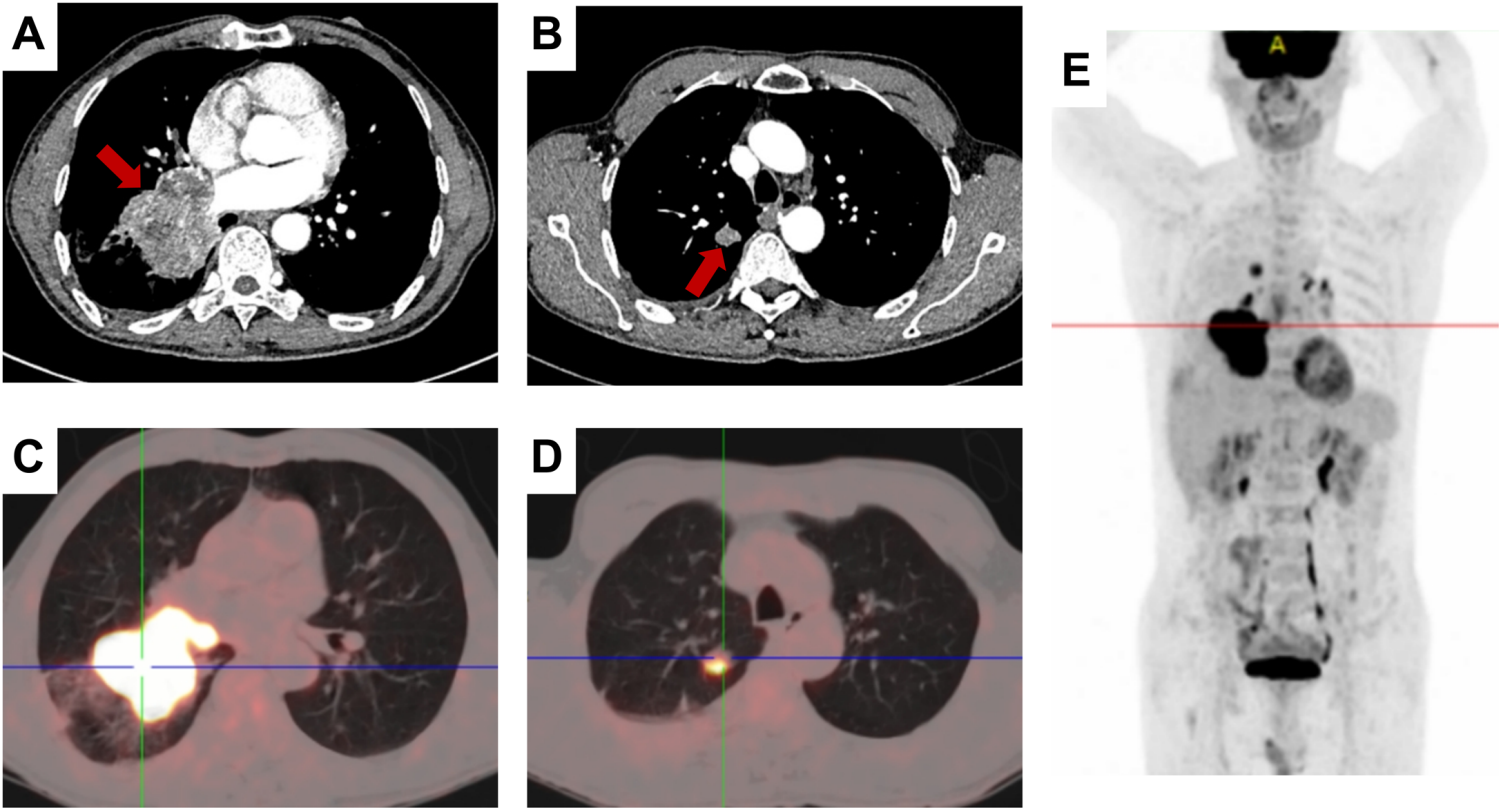
Radiological findings. **(A, B)** Contrast-enhanced chest CT showing a mass in the right lower lung lobe; the red arrow indicating the tumor region. **(C–E)** 18F-FDG PET/CT showing FDG avidity and extent of the pulmonary tumoral sites

In October 2023, a 67-year-old man was referred to our hospital with a malignant tumor in the lung. He had no past history of any disease, and there was no occupational exposure to gas, dust, or fumes. Nearly 1 month ago, he went to a local hospital with a cough, expectoration, and blood in the sputum; a chest computed tomography (CT) scan showed a mass in the lower lobe of the right lung. The admission physical examination in our hospital was normal and his testicles were equal in size with no lumps or nodules. Contrast-enhanced chest CT showed a 73 mm mass in the lower lobe of the right lung, accompanied by obstructive pneumonia in the right lung, cancer lymphangitis in the right lower lung, multiple metastases in the bilateral lungs, and possible right hilar lymph node metastasis (**Figures 1A, B**). 18F-fluorodeoxyglucose positron emission tomography/computed tomography (18F-FDG-PET/CT) imaging showed intense uptake in a 66.2 mm soft tissue mass in the lower lobe of the right lung and 11.7 mm or 6.1 mm nodules in the upper lobes of the bilateral lungs and right hilar lymph nodes (**Figures 1C–E**). Other scans of the abdomen, pelvis, brain, and bone showed no abnormalities.

**Figure 2:**
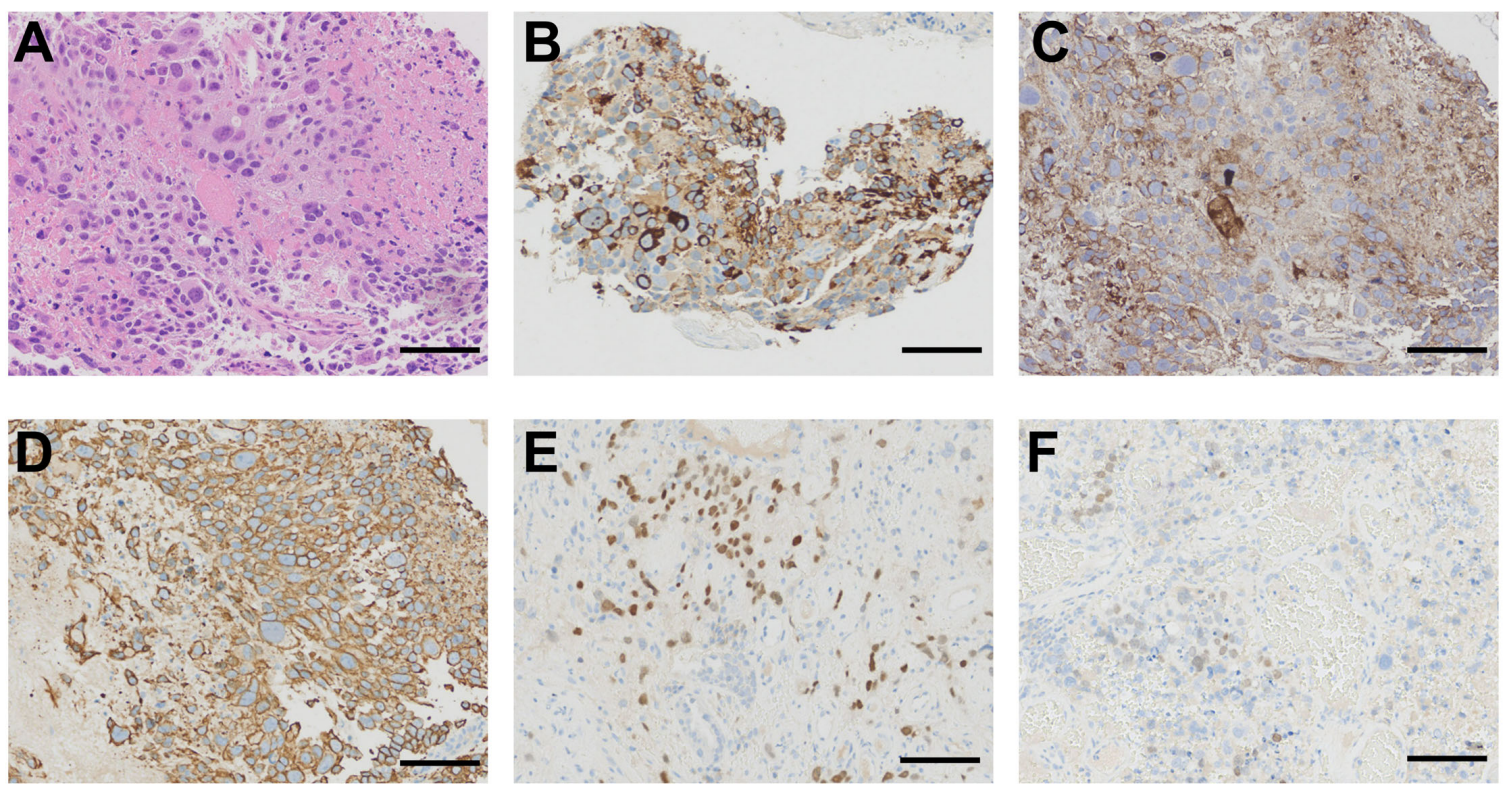
Histological findings. **(A)** Primary lesion obtained from bronchoscopy showing a malignant tumor composed of large tumor cells with enlarged hyperchromatic nuclei and abundant eosinophilic cytoplasm (hematoxylin and eosin staining). **(B)** Immunohistochemical staining of the primary lesion showing a strong positive reaction for HCG. **(C)** Immunohistochemical staining for PD-L1 revealing 70% positive cells. **(D)** Immunohistochemical staining for CK-pan (AE1/AE3) showing strong positivity. **(E)** Immunohistochemical staining for GATA-3 showing partial positivity. **(F)** Immunohistochemical staining for SALL4 showing partial positivity. Scale bar= 100μm.

The transbronchial biopsy revealed a mass in the right middle bronchus. A microscopic pathological examination indicated poorly differentiated carcinoma with large areas of hemorrhage and necrosis. Our pathologist further analyzed the hematoxylin and eosin staining of the primary lesion and found that the malignant tumor consisted of multinucleated syncytiotrophoblasts, cytotrophoblasts, and intermediate trophoblasts (**Figure 2A**). The immunohistochemical results were as follows: TTF-1 (–), Napsin A (–), P63(partially +), P40(partially +), CK-pan(AE1/AE3)(+), BRG1(+, not missing), INSM1(-), CgA(-), Syn(-), Ki-67(+, 50%-60%), INI-1(+, not missing), NUT(-), PD-L1(22C3)(+, TPS=70%), SALL4(partially +), GATA-3(partially +), HCG(partially +), CD10(+), Inhibina(-), and P53(+, wild-type) (**Figure 2**).

**Figure 3:**
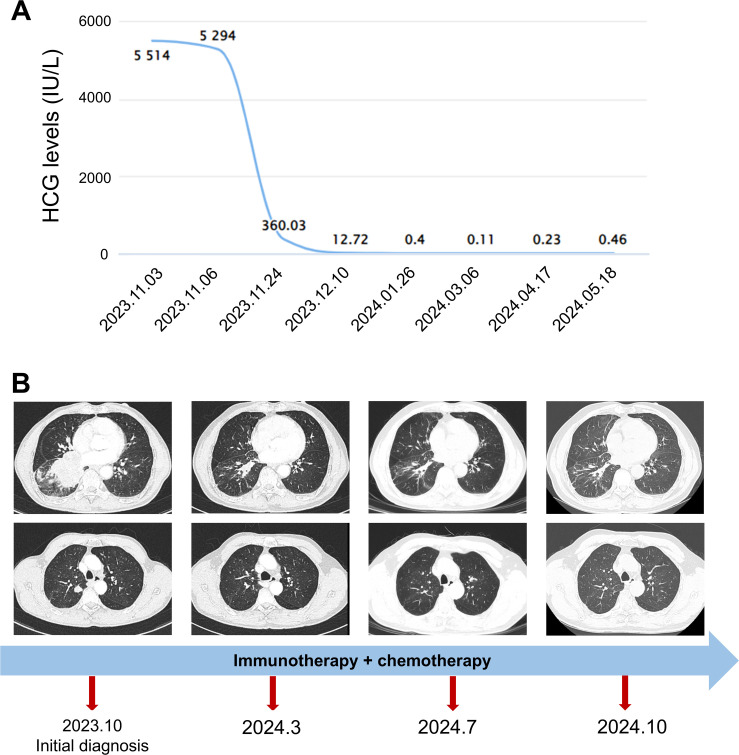
The change in serum HCG levels and chest CT finding. **(A)** The changes in serum HCG levels. **(B)** The timeline and changes in the lung mass and nodules revealed by chest CT. The start date of chemoimmunotherapy was 2023.11.06.

The patient’s serum HCG level was high at 5,514 IU/L. After consultation with the pathology and oncology departments, the patient was diagnosed with PPC. He received chemotherapy combined with immunotherapy (cisplatin or etoposide, paclitaxel, and sintilimab). Within 3 months of starting therapy, the patient’s serum HCG dropped to 0.40 IU/L (**Figure 3A**). A chest CT scan showed a significant reduction in the mass in the right lung after 4 months (**Figure 3B**). The patient received treatment and follow-up examinations (serum HCG, thorax CT) in our hospital, and has not shown a recurrence of the disease up to now. The follow-up period has been 12 months thus far.

## Methods

We searched the keyword “primary pulmonary choriocarcinoma” in PubMed and collected all patients diagnosed with PPC that were published in the English literature until 1 October 2024. The earliest reports were from 1953 and the latest results were from 2024. All results were checked for eligibility. Only reports with informative abstracts or full text were included. In total, 36 male cases were identified. We extracted the data of the patients including sex, age, initial symptoms, tumor location, metastasis, therapy, and survival time.

Overall survival (OS) was the primary endpoint and was defined as the time from diagnosis to death from any cause or last follow-up time. Survival curves were depicted using the Kaplan–Meier method and compared using the stratified log-rank test. The simultaneous effects of prognostic factors on survival were estimated by a Cox proportional hazards model. Graphpad Prism 7.0 software was used to perform a statistical analysis of the risk factors. A P-value of 0.05 or less denoted a statistically significant difference. All statistical tests were two-sided.

## Literature review and discussion

As a highly malignant trophoblastic tumor, choriocarcinoma produces HCG and frequently originates from the gonads including the ovaries and testicles. Extragonadal choriocarcinoma is uncommon and reported cases arose in the retroperitoneum, mediastinum, vicinity of the pineal body, lungs, brain, alimentary tract, liver, kidneys, and urinary bladder (2, 6). PPC is an extremely rare tumor with 68 patients for whom medical record information was available (2). Our study identified 36 male cases with informative abstracts or full text from 1953 to 2024 (**Supplementary Table 1**).

The clinical characteristics of PPC patients are diverse. The analysis results of the 36 male cases in this study revealed the common symptoms included a cough (11/36, 30.6%), hemoptysis (11/36, 30.6%), chest pain (9/36, 25.0%), gynecomastia (7/36, 19.4%), dyspnea (6/36, 16.7%), and weight loss (5/36, 13.9%). There are also some patients who had no symptoms (6/36, 16.7%) (**Supplementary Table 2**). Furthermore, the feminization found in the male patients, including libido loss and gynecomastia, was due to elevated HCG levels. However, the HCG test in male patients is always neglected in clinical practice, which causes male primary choriocarcinoma to be often missed or misdiagnosed. It is difficult to identify PPC at the early stages, leading to a poor prognosis and treatment failure (7). Therefore, early diagnosis and therapy are critical. The diagnosis of PPC is based on the histological and immunohistochemical features. In the present case, the hematoxylin and eosin staining of the primary lesion showed a malignant tumor consisting of multinucleated syncytiotrophoblasts, cytotrophoblasts, and intermediate trophoblasts. The immunohistochemical profile of germ cell origin (such as HCG, SALL4, and GATA-3) was positive (**Figure 2**). A further test result of the elevation in serum HCG level led to the diagnosis of PPC.

**Figure 4:**
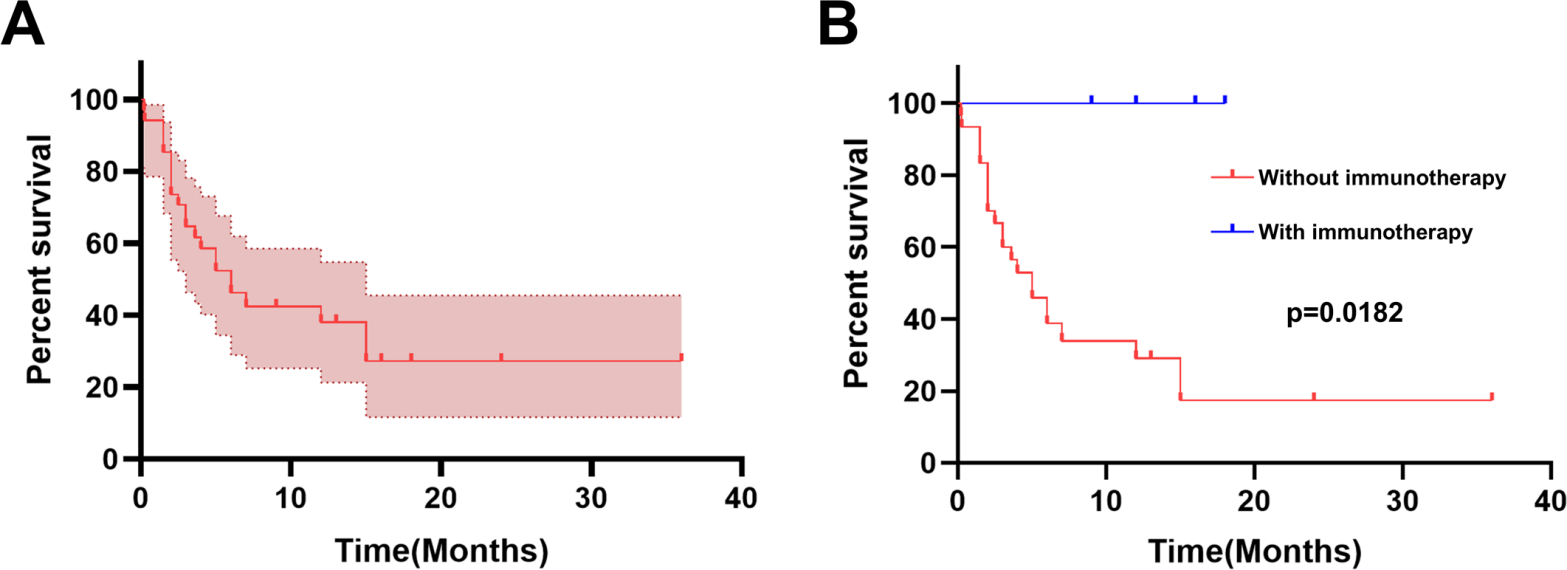
Kaplan−Meier curve for survival. **(A)** The Kaplan−Meier curve for overall survival (N = 34). **(B)** The survival percentage in PPC was calculated using the Kaplan−Meier method stratified by with or without immunotherapy.

It has been reported that gestational choriocarcinoma responds well to chemotherapy, even when detected at a late stage. However, PPC responds poorly to chemotherapy and progresses rapidly. PPC patients have a very poor prognosis with a dismal 5-year survival rate (less than 5%) (8). According to our literature review, the 1-year survival rate among the male cases with obtained survival time was 37.66% (**Figure 4A**). Thus far, there are no clinical guidelines for the treatment of PPC. Surgery combined with chemotherapy seems to be the most appropriate treatment according to earlier case reports (2). Analysis of previous cases shows that the chemotherapy regimens commonly used in PPC treatment were mostly based on female choriocarcinoma or male germ cell tumors, the most common chemotherapy regimens for PCC included the PEB regimen (cisplatin, etoposide, and bleomycin), EMA-CO regimen (etoposide, methotrexate, actinomycin D/cyclophosphamide, and vincristine), EMA-EP regimen (etoposide, methotrexate, actinomycin-D/etoposide, and cisplatin), and so on. However, the prognosis is not ideal.

Recently, ICIs have been promising for the treatment of choriocarcinoma since they can produce durable responses against multiple tumor types and provide long-term survival benefits, which gives them the potential to treat chemotherapy-resistant choriocarcinoma (4). Reviewing the previous studies in the literature reveals that most choriocarcinoma specimens exhibited diffuse and intense PD-L1 immunoreactivity in syncytiotrophoblasts (3). In some choriocarcinoma cases, ICIs elicited a long-term antitumor response after failed chemotherapy (9–12). The introduction of ICI therapy in PPC has achieved good results in clinical settings (**Supplementary Table 3**). A 60-year-old PPC patient with positive PD-L1 (more than 50%, 22C3) was given nivolumab after chemotherapy and obtained a partial response for 11 months (10). Devos et al. found that the tumor proportion score of PD-L1 expression was 10% in a 65-year-old PPC patient, and treated him with a chemoimmunotherapy regimen including carboplatin, etoposide, and pembrolizumab with good partial response (13). Iso et al. reported a 72-year-old male, who received a combination of carboplatin, paclitaxel, nivolumab, and ipilimumab, and achieved a partial response. Interestingly, the PD-L1 expression of this patient was negative (0%) (4). In our case, the tumor had PD-L1 expression with a tumor proportion score of 70%. Therefore, we combined chemotherapy with sintilimab to treat our patient. Encouragingly, the patient achieved a good partial response for 12 months.

We calculated the overall survival percentage in 34 male PPC patients (for whom survival time could be obtained) using the Kaplan−Meier method stratified by immunotherapy. As shown in **Figure 4B**, it is significantly different (log-rank test, P = 0.0182) when comparing overall survival between those who received immunotherapy and those without immunotherapy. Patients treated without ICIs had a 1-year survival rate of 29.12%, while all 4 patients who received immunotherapy survived during the follow-up period. These results indicate that ICIs are a powerful treatment for PPC. It is worth noting that the sample size of patients who received immunotherapy is too small to make a meaningful comparison. However, the good overall prognosis of these cases could preserve a patient’s hope. ICIs may be a promising therapy option for PPC patients.

## Conclusion

PPC is a rare disease in men with high aggressivity and poor response to chemotherapy. There is no established standard treatment for it up to now. Our literature review revealed that the application of ICIs combined with chemotherapy could prolong the survival time of PPC patients, which could preserve the patient’s hope. ICIs may be a promising therapy option for PPC patients.

## Data Availability

The original contributions presented in the study are included in the article/**Supplementary Material**. Further inquiries can be directed to the corresponding author.
