# Supplementary material for: Combination chemotherapy with sintilimab for treatment of a male patient with primary pulmonary choriocarcinoma: a case report and literature review
Source: Front Immunol. 2025 Jan 23;16:1523316. doi: 10.3389/fimmu.2025.1523316 (PMC11799555; doi:10.3389/fimmu.2025.1523316)
Supplement: Supplementary file 1 [file Table1.docx]

**Supplementary Table 1. Summary of the male PPC cases reported in literature**

| Age (Year) | | Initial symptom(s) | Location | Tumor size (cm) | Metastasis | Therapy | Survival  (months) | Ref |
| --- | --- | --- | --- | --- | --- | --- | --- | --- |
| 45 | Gynecomastia | | LUL | 7 | Lung, liver, kidney, spleen | Radiotherapy | 5 | (1) |
| 57 | Cough, gynecomastia | | RLL | 10 | Brain | Radiotherapy | 12 | (1) |
| 63 | Cough, dyspnea, gynecomastia | | Bilateral | 12 | Lung, pleura, diaphragm, pericardium, lymph nodes | Chemotherapy | 2 | (2) |
| 27 | | Chest pain | RUL | 4 | None | Operation, chemotherapy | >24 | (3) |
| 41 | | Gynecomastia | RUL | 5 | None | Operation, chemotherapy | NA | (4) |
| 37 | | Upper abdomen pain, gynecomastia | Bilateral | 7 | Lung, liver, brain, spleen, kidney, intestine | Chemotherapy | 2.5 | (5) |
| 67 | | Hemoptysis | LUL | 5 | None | Operation | >36 | (6) |
| 71 | | Dyspnea, chest pain, upper abdomen pain, gynecomastia | RUL | 3 | Lung, liver, kidney, adrenal gland, bone marrow and lymph nodes | Others | 6 | (7) |
| 37 | | Cough, chest pain, hemoptysis, fever | RUL | 11 | Lung and brain | Operation, Chemotherapy and radiotherapy | 15 | (8) |
| 51 | | Cough, weight loss | LUL | 15 | Lung, brain, liver, spleen | Radiotherapy | 0.2 | (9) |
| 61 | | Hemoptysis, chest pain | LUL | 6 | Lung | Operation, radiotherapy, chemotherapy | 1.5 | (10) |
| 0.33(4m) | | Pubic hair, enlarged penis, pigmented scrotal skin | RLL | 6.5 | Brain | Operation, chemotherapy, radiotherapy, | 7 | (11) |
| 69 | | Hemoptysis, weight loss | Bilateral | 18 | Lung, liver, adrenals, lymph nodes | Chemotherapy | 1.5 | (12) |
| 69 | | None | LUL | 5 | None | Operation, chemotherapy | >6 | (13) |
| 60 | | None | R | NA | Lung, brain | Chemotherapy | 5 | (14) |
| 61 | | Hemoptysis | RUL | NA | Lung | Operation, chemotherapy | >6 | (15) |
| 23 | | Hemoptysis, dyspnea | Bilateral | NA | NA | Chemotherapy | 0.26 | (16) |
| 46 | | Cough, dyspnea, chest pain, weight loss | RUL | 18 | None | Operation, chemotherapy | >6 | (17) |
| 77 | | None | LUL | 5.5 | Lungs, liver, spleen, heart, adrenal glands,  kidneys, vertebrae, thyroid gland, lymph nodes, pancreas | None | 2 | (18) |
| 48 | | Cough, dyspnea, weight loss | Bilateral | NA | liver, brain | Chemotherapy, Operation | 3 | (19) |
| 33 | | Chest pain | Bilateral | NA | None | Chemotherapy | >3 | (20) |
| 59 | | Hemoptysis | LUL | 2.8 | None | Operation | 1.5 | (21) |
| 67 | | None | L | 9 | None | Operation, chemotherapy (PEB) | >13 | (22) |
| 70 | | Cough | RUL | 3.8 | None | Operation | >24 | (23) |
| 71 | | Cough, hemoptysis | RML | 1.6 | None | Operation | 3 | (24) |
| 53 | | Cough, chest pain | LUL | 6 | Lung, brain, stomach | Chemoradiotherapy, operation | 15 | (25) |
| 70 | | Hemoptysis | R | 10 | Brain, liver, adrenal gland, kidneys | None | 2 | (25) |
| 77 | | Hemoptysis | LUL | 5.5 | Lung, spleen, pancreas | None | 2 | (25) |
| 77 | | Tumble | R | 3.5 | None | None | 4 | (25) |
| 60 | | Headache, ataxia, dizziness | RUL, RML | NA | Brain | Chemotherapy, immunotherapy | >16 | (26) |
| 42 | | Chest pain, weight loss | RUL | 15 | Lung | Operation, chemotherapy (EMA-CO) | 6 | (27) |
| 65 | | None | LUL | 4.2 | None | Operation | NA | (28) |
| 69 | | Dyspnea, chest pain, shoulder pain | LUL | 2 | Lung, liver | None | 3.6 | (29) |
| 72 | | None | LUL | 1.8 | None | Operation, chemotherapy, immunotherapy | >18 | (30) |
| 65 | | Cough, gynecomastia, libido loss, agitation | Bilateral | 13 | Brain | Chemotherapy, immunotherapy | >9 | (31) |
| 67 | | Cough, hemoptysis | RLL | 7.3 | Lung | Chemotherapy, immunotherapy | >12 | Our case |

RLL, right lower lobe. RUL, right upper lobe. RML, right middle lobe. R, right. LUL, left upper lobe.

**Supplementary Table 2. Summary of** **clinical characteristics of 36 PPC cases**

| **Characteristics** | **N=36** | **%** |
| --- | --- | --- |
| **Age(years)**  <40  ≥40 | 6  30 | 16.7  83.3 |
| **Initial symptoms**  Cough  Hemoptysis  Chest pain  Gynecomastia  Dyspnea  Weight loss  Abdomen pain  None | 11  11  9  7  6  5  2  6 | 30.6  30.6  25  19.4  16.7  13.9  5.6  16.7 |
| **Lesion location**  L  R | 13  16 | 36.1  44.5 |
| Bilateral | 7 | 19.4 |
| **Tumor size (cm)**  ≤5  ＞5  NA | 12  18  6 | 33.3  50.0  16..7 |
| **Treatment**  With immunotherapy  Without immunotherapy | 4  32 | 11.1  89.9 |

**Supplementary Table 3. Reported cases of male PPC treated with immunotherapy**

| **Age (years)** | **PD-L1 (%)** | **Treatment** | **Best response** | **Survival time (months)** | **Ref** |
| --- | --- | --- | --- | --- | --- |
| 60 | >50% | Nivolumab | PR | >16 | (26) |
| 72 | 0% | Operation+ chemotherapy+ nivolumab, ipilimumab | PR | >18 | (30) |
| 65 | 10% | Chemotherapy+ pembrolizumab | PR | >9 | (31) |
| 67 | 70% | Chemotherapy+ sintilimab | PR | >12 | Our case |

PR, partial response.

**References**

1. Hayakawa K, Takahashi M, Sasaki K, Kawaoi A, Okano T. Primary Choriocarcinoma of the Lung: Case Report of Two Male Subjects. *Acta Pathol Jpn* (1977) 27(1):123-35. Epub 1977/01/01.

2. Hattori M, Imura H, Matsukura S, Yoshimoto Y, Sekita K, Tomomatsu T, et al. Multiple-Hormone Producing Lung Carcinoma. *Cancer* (1979) 43(6):2429-37. Epub 1979/06/01. doi: 10.1002/1097-0142(197906)43:6<2429::aid-cncr2820430639>3.0.co;2-i.

3. Kalla AH, Voss EC, Jr., Reed RJ, 3rd. Primary Choriocarcinoma of the Lung: (a Case Report). *W V Med J* (1980) 76(10):261-3. Epub 1980/10/01.

4. Whitcomb RW, Schimke RN, Kyner JL, Lukert BP, Johnson DC. Endocrine Studies in a Male Patient with Choriocarcinoma and Gynecomastia. *Am J Med* (1986) 81(5):917-20. Epub 1986/11/01. doi: 10.1016/0002-9343(86)90369-4.

5. Endou T, Ueno H, Okada H, Yano S, Noda M. [Primary Choriocarcinoma of the Lung (a Case Report of Male Subject and Review of the Literature)]. *Nihon Naika Gakkai Zasshi* (1988) 77(9):1404-9. Epub 1988/09/01. doi: 10.2169/naika.77.1404.

6. Zapatero J, Bellon J, Baamonde C, Aragoneses FG, Cubillo J, Orusco E, et al. Primary Choriocarcinoma of the Lung. Presentation of a Case and Review of the Literature. *Scand J Thorac Cardiovasc Surg* (1982) 16(3):279-81. Epub 1982/01/01. doi: 10.3109/14017438209101063.

7. Adachi H, Aki T, Yoshida H, Yumoto T, Wakahara H. Combined Choriocarcinoma and Adenocarcinoma of the Lung. *Acta Pathol Jpn* (1989) 39(2):147-52. Epub 1989/02/01. doi: 10.1111/j.1440-1827.1989.tb01493.x.

8. Sridhar KS, Saldana MJ, Thurer RJ, Beattie EJ. Primary Choriocarcinoma of the Lung: Report of a Case Treated with Intensive Multimodality Therapy and Review of the Literature. *J Surg Oncol* (1989) 41(2):93-7. Epub 1989/06/01. doi: 10.1002/jso.2930410208.

9. Sullivan LG. Primary Choriocarcinoma of the Lung in a Man. *Arch Pathol Lab Med* (1989) 113(1):82-3. Epub 1989/01/01.

10. Durieu I, Berger N, Loire R, Gamondes JP, Guillaud PH, Cordier JF. Contralateral Haemorrhagic Pulmonary Metastases ("Choriocarcinoma Syndrome") after Pneumonectomy for Primary Pulmonary Choriocarcinoma. *Thorax* (1994) 49(5):523-4. Epub 1994/05/01. doi: 10.1136/thx.49.5.523.

11. Otsuka T, Ohshima Y, Sunaga Y, Nagashima K. Primary Pulmonary Choriocarcinoma in a Four Month Old Boy Complicated with Precocious Puberty. *Acta Paediatr Jpn* (1994) 36(4):404-7. Epub 1994/08/01. doi: 10.1111/j.1442-200x.1994.tb03210.x.

12. Toda S, Inoue Y, Ishino T, Yonemitsu N, Terayama K, Miyabara S, et al. A Rare Case of Primary Pulmonary Choriocarcinoma in a Male: Immunohistochemical Detection for Human Chorionic Gonadotropin, Epidermal Growth Factor (Egf) and Egf-Receptor. *Endocr J* (1995) 42(5):655-9. Epub 1995/10/01. doi: 10.1507/endocrj.42.655.

13. Canver CC, Voytovich MC. Resection of an Unsuspected Primary Pulmonary Choriocarcinoma. *Ann Thorac Surg* (1996) 61(4):1249-51. Epub 1996/04/01. doi: 10.1016/0003-4975(95)01158-7.

14. Ikura Y, Inoue T, Tsukuda H, Yamamoto T, Ueda M, Kobayashi Y. Primary Choriocarcinoma and Human Chorionic Gonadotrophin-Producing Giant Cell Carcinoma of the Lung: Are They Independent Entities? *Histopathology* (2000) 36(1):17-25. Epub 2000/01/13. doi: 10.1046/j.1365-2559.2000.00789.x.

15. Chen F, Tatsumi A, Numoto S. Combined Choriocarcinoma and Adenocarcinoma of the Lung Occurring in a Man: Case Report and Review of the Literature. *Cancer* (2001) 91(1):123-9. Epub 2001/01/10. doi: 10.1002/1097-0142(20010101)91:1<123::aid-cncr16>3.0.co;2-3.

16. Tsai JR, Chong IW, Hung JY, Tsai KB. Use of Urine Pregnancy Test for Rapid Diagnosis of Primary Pulmonary Choriocarcinoma in a Man. *Chest* (2002) 121(3):996-8. Epub 2002/03/13. doi: 10.1378/chest.121.3.996.

17. Okur E, Halezeroglu S, Somay A, Atasalihi A. Unusual Intrathoracic Location of a Primary Germ Cell Tumour. *Eur J Cardiothorac Surg* (2002) 22(4):651-3. Epub 2002/09/26. doi: 10.1016/s1010-7940(02)00390-1.

18. Yamamoto S, Tanaka H, Takeo H, Yasuda K, Mastukuma S. Primary Pulmonary Choriocarcinoma Combined with Adenocarcinoma. *Pathol Int* (2006) 56(7):402-7. Epub 2006/06/24. doi: 10.1111/j.1440-1827.2006.01977.x.

19. Hadgu A, Tindni A, Panda M. Primary Pulmonary Choriocarcinoma in a Male. *BMJ Case Rep* (2010) 2010. Epub 2010/01/01. doi: 10.1136/bcr.02.2010.2712.

20. Das S, Cherian SV, Das N, Haq EU, Hamarneh WA, Lenox R, et al. A 33-Year-Old Man with Chest Pain, Left Upper Lobe Mass, Pulmonary Nodules, and Bilateral Bullae. *Chest* (2012) 142(4):1058-62. Epub 2012/10/04. doi: 10.1378/chest.12-0011.

21. Takahashi T, Kobayashi R. Choriocarcinoma Syndrome after Resection of Primary Pulmonary Choriocarcinoma: Report of a Case. *Surg Case Rep* (2016) 2(1):122. Epub 2016/11/04. doi: 10.1186/s40792-016-0227-5.

22. Zhu R, Jia C, Yan J, Luo Y, Huo Z. Primary Pulmonary Choriocarcinoma in a Male That Was Successfully Diagnosed and Treated: A Case Report and Review of the Literature. *Medicine (Baltimore)* (2016) 95(52):e5693. Epub 2016/12/30. doi: 10.1097/md.0000000000005693.

23. Kamata S, Sakurada A, Sato N, Noda M, Okada Y. A Case of Primary Pulmonary Choriocarcinoma Successfully Treated by Surgery. *Gen Thorac Cardiovasc Surg* (2017) 65(6):361-4. Epub 2016/05/30. doi: 10.1007/s11748-016-0666-8.

24. Ma Y, Wang C, Sun PL, Zhu Y, Huang ZK, Jin SX. A Case of Male Primary Pulmonary Choriocarcinoma. *Chin Med J (Engl)* (2018) 131(24):3001-3. Epub 2018/12/13. doi: 10.4103/0366-6999.247205.

25. Matsukuma S, Obara K, Utsumi Y, Miyai K, Takeo H, Oshika Y, et al. Focal Positivity of Immunohistochemical Markers for Pulmonary Squamous Cell Carcinoma in Primary Pulmonary Choriocarcinoma: A Histopathological Study. *Oncol Lett* (2018) 16(6):7256-63. Epub 2018/12/14. doi: 10.3892/ol.2018.9525.

26. Ochi M, Miyamoto S, Terada Y, Furuhata Y, Awano N, Izumo T, et al. The Significant Antitumor Activity of Nivolumab in Lung Adenocarcinoma with Choriocarcinomatous Features. *Intern Med* (2018) 57(12):1773-7. Epub 2018/02/13. doi: 10.2169/internalmedicine.0002-17.

27. Nguyen HTT, Hoang HH, Le ATV. A Case Report of Primary Pulmonary Choriocarcinoma in a Man: Successful Combination of Surgery and Chemotherapy. *Case Rep Oncol* (2020) 13(2):923-8. Epub 2020/09/05. doi: 10.1159/000508744.

28. Zhang X, Ding B, Chen L, Huang X, Zhang K, Wang Z, et al. Primary Pulmonary Choriocarcinoma in Male: Report a Case with Genetic Testing and Review of the Literature. *Transl Cancer Res* (2022) 11(6):1844-9. Epub 2022/07/16. doi: 10.21037/tcr-21-2627.

29. Iwasaki K, Watanabe K, Kimura H, Yano S. Utility of Beta-Human Chorionic Gonadotropin in Pleural Effusions: Report of an Autopsy Case of a Male Patient with Primary Pulmonary Choriocarcinoma. *Clin Case Rep* (2022) 10(12):e6663. Epub 2022/12/10. doi: 10.1002/ccr3.6663.

30. Iso H, Hisakane K, Terashi N, Mikami E, Matsuki S, Sonokawa T, et al. A Remarkable Response to Combination Chemotherapy with Nivolumab and Ipilimumab in a Patient with Primary Pulmonary Choriocarcinoma: A Case Report. *Transl Cancer Res* (2023) 12(8):2212-8. Epub 2023/09/13. doi: 10.21037/tcr-23-221.

31. Devos B, Willemse C, Sterckx M, Debruyne J, Stappaerts I, Van den Mooter T, et al. Beta-Hcg Secretion by a Pulmonary Choriocarcinoma in a Male Patient. *Case Rep Oncol Med* (2024) 2024:8731806. Epub 2024/02/05. doi: 10.1155/2024/8731806.
